# Lexical References to Sensory Modalities in Verbal Descriptions of People and Objects by Congenitally Blind, Late Blind and Sighted Adults

**DOI:** 10.1371/journal.pone.0044020

**Published:** 2012-08-30

**Authors:** Valérie Chauvey, Yvette Hatwell, Bertrand Verine, Gwenael Kaminski, Edouard Gentaz

**Affiliations:** 1 Laboratoire de Psychologie et NeuroCognition (UMR CNRS 5105), Université Pierre Mendès-France, Grenoble, France; 2 Laboratoire Praxiling (UMR CNRS 5267), University Paul-Valéry, Montpellier, France; 3 Laboratoire CLLE-LTC, (UMR CNRS 5263), Université de Toulouse Le Mirail, Toulouse, France; French National Centre for Scientific Research, France

## Abstract

**Background:**

Some previous studies have revealed that while congenitally blind people have a tendency to refer to visual attributes (‘verbalism’), references to auditory and tactile attributes are scarcer. However, this statement may be challenged by current theories claiming that cognition is linked to the perceptions and actions from which it derives. Verbal productions by the blind could therefore differ from those of the sighted because of their specific perceptual experience. The relative weight of each sense in oral descriptions was compared in three groups with different visual experience Congenitally blind (CB), late blind (LB) and blindfolded sighted (BS) adults.

**Methodology/Principal Findings:**

Participants were asked to give an oral description of their mother and their father, and of four familiar manually-explored objects. The number of visual references obtained when describing people was relatively high, and was the same in the CB and BS groups (“verbalism” in the CB). While references to touch were scarce in all groups, the CB referred to audition more frequently than the LB and the BS groups. There were, by contrast, no differences between groups in descriptions of objects, and references to touch dominated the other modalities.

**Conclusion/Significance:**

The relative weight of each modality varies according to the cognitive processes involved in each task. Long term memory, internal representations and information acquired through social communication, are at work in the People task, seem to favour visual references in both the blind and the sighted, whereas the congenitally blind also refer often to audition. By contrast, the perceptual encoding and working memory at work in the Objects task enhance sensory references to touch in a similar way in all groups. These results attenuate the impact of verbalism in blindness, and support (albeit moderately) the idea that the perceptual experience of the congenitally blind is to some extent reflected in their cognition.

## Introduction

Available research on the oral language of blind people mainly concerns the acquisition of language in infancy [1]–[3]. For many years, numerous studies have concentrated on the question of verbalism in school-age children and more rarely in adults. Recently, there has been a renewal of interest in the language of the blind, as discussed below, due to the success of the theories of “embodied” or “grounded” cognition” [4], [5].

Verbalism refers to the fact that blind people often use words which have no sensory basis, because they express purely visual experiences such as color and light, or refer to other attributes of objects which cannot be perceived by the blind. According to Cutsforth [6], [7], this particularity is specific to the blind and seriously impairs their cognitive development, because their blurred thinking is disconnected from perceptual experience. Cutsforth presented orally a series of words to blind children, and asked them to enumerate the attributes of these objects. The congenitally blind evoked visual attributes in 48% of their answers, whereas the late blind evoked visual attributes in 65% of their answers. Similarily, von Tetzchner and Martinsen [8] reported higher rates of verbalism in blind than in sighted children (cf. also [9]).

Further studies have consistently attenuated Cutsforth’s (1951) point of view. In a word association test, Nolan [10] found no difference between blind and sighted children. Harley [11] compared the definitions of words given by blind 7 to 14 year-olds with their identification of the physical objects represented by these words. Harley made a distinction between visual verbalism (the use of words referring to vision) and global verbalism, in which the child identified words which were not based on his/her own perceptual experience. While purely visual verbalism was relatively rare, global scores of verbalism were high and correlated negatively with chronological age and IQ. According to Harley, the relationship between verbalism and intellectual level means that the youngest children and those with the lowest cognitive level produced more verbalisms than the others. It should, however, be noted that the method Harley [11] used to evaluate verbalism was somewhat questionable as it involved calculating the number of words correctly defined verbally while they were not physically identified. But the task of identification did include many real miniaturized objects (figurines, small toys representing large objects of the environment, etc.). We know that identification of these miniaturized objects is often difficult for the blind [1]. For example, failure to identify a figurine of a cow obviously does not mean that the child has no perceptual experience of cows.

The decrease in verbalism with age observed by Harley [11] was contradicted by a more recent work by Rosel, Caballer, Jara, and Oliver [12] using a different method (verbal productions of free stories based on a number of stimulus words given at the beginning of the task). They observed no differences between blind and sighted children (6–14 years). They revealed however a highly significant age effect, with the number of verbalisms increasing with age in both populations. This indicates that, as the child grows and progressively integrates all the aspects of adult language, his own verbal communication becomes less specific to blindness and increasingly similar to that of sighted people.

Anderson and Olson [13] compared in children aged from 3 to 10 verbal definitions of objects “tangible” (that could be grasped by the hand) or less tangible (palpable but too large to be held in the hand (*car, tree*, etc.). No differences emerged between blind and sighted children, but the definitions given by the blind to the most tangible words were more functional (*“what is it used for?”*) and less perceptually-based. As expected, the words corresponding to the less tangible objects were described with fewer words than the more tangible ones, but in all cases, the tactile information on these objects was dominant. The authors concluded that the language of blind children expressed the concepts they have built from their specific perceptual experience.

In the same vein, a very recent study by Vinter and his colleagues [14] showed than when blind and normally sighted children aged 6–14 years were asked to define familiar words denoting concrete animate or inanimate objects (tangible or not tangible, manipulable or not, etc), more tactile and auditory characteristics were expressed by the blind whereas the sighted relied more on visual perceptions.

Although Millar [15] contested the negative effect of verbalism on cognition, she demonstrated clearly that pure visual verbalism does exist in blind children. She presented a series of word-pairs verbally to sighted and congenitally blind children aged between 8 and 13. Each pair was composed of a noun and an adjective, and the adjective could refer to a visual (color), tactile, auditory or spatial property. In half the cases, the noun-adjective coupling was appropriate (*ball-round*), and in the second half it was inappropriate (*snow-black*). The child was asked to say if the coupling in each pair was correct or incorrect. Very few errors were observed in either the sighted (2%) or the congenitally blind (4%) groups. In the latter group, errors were due to the youngest children, whereas from 10 years old on, the congenitally blind performed as well as the sighted children for the appropriate and inappropriate pairs. The only difference between sighted and blind children concerned response latency, which was longer for inappropriate pairings in the blind group. This study showed, therefore, that even congenitally blind children are able to correctly identify the association of a noun and a color they have never perceived.

Millar [16] rightly rejected the idea that what is called “verbalism” is detrimental to cognitive development. Even in sighted people, knowledge very often derives from indirect sources and not from direct perceptual experiences. It is not, for example, necessary to have seen a fairy or a dragon or to believe in their existence in order to understand what the words mean.

This analysis of previous literature suggests that although blind children manifest verbalism, they refer to their specific auditory and tactile perceptual experiences more often in their verbal productions than in the verbal productions of the sighted. No experimental works are available on verbalism in blind adults. An informal observation made recently by one of us suggested, however, that verbalism remained high in blind adults. This tendency appeared in the analysis of a sample of written texts produced by visually impaired adults during the competition organised by Bertrand Vérine in Paris in 2009 to celebrate the 200^th^ anniversary of the birth of Louis Braille. In this examination entitled “Saying the non-visual” (*“Dire le non-visuel”)*, the participants were asked “to relate a sensory experience other than a visual one, or one which involved the description of characters, objects or places through auditory, tactile, olfactory or taste perception”. The written productions obtained in these conditions continued to reveal frequent use of visual words (despite instructions to the contrary), as well as less frequent use of some tactile, auditory and olfactory words (for details, see http://www.fafdirelenonvisuel.org [17]).

Today, the study of language in the blind has been renewed by the development of theories of “embodied cognition” [4], [5]. These theories firmly reject the notion of amodal cognition, and claim by contrast that knowledge is always bodily grounded (even the more abstract concepts), i.e. it relies on the perceptions and actions from which it derives. If knowledge is, as these theories assume, actually body-based then we ought to find more evidence of this bodily implication in blind people, due to their constant use of touch (and other sensory modalities) in order to compensate for visual deprivation. Unlike vision which functions by tele-reception (the perception of distant objects), touch receptors are activated only when the skin comes into direct contact with an object. The perceptual field is therefore very narrow, and specific exploratory movements are necessary to perceive the whole object [18–19–20]. The implication of the whole body (perceptions and actions) may, therefore, be higher in the acquisition of knowledge by the blind and, as a result, the verbal productions of blind people may contain more sensory references to these perceptions and actions than those of sighted people. The results already quoted obtained by Anderson and Olson [13] may support this view. Similarly and more recently, Sanchez, Faber, and D’Angiulli [21] interpreted their findings concerning language and drawing in blind children with reference to these embodied theories of cognition.

To sum up, it is understandable that congenitally blind adults often use visual references in their language, even when their blindness occurred early in age. These blind peoples’ lives are totally integrated with the sighted way of life. They read books written by and for the sighted, and they enjoy the same films. The crucial question, therefore, concerns their references to the other sensory modalities: are these references really very scarce? Or, as can be assumed from theories of grounded cognition, does the intensive haptic and auditory perceptual experience of the blind appear in their verbal productions more than in those of sighted people?

Because the literature yields contradictory results and there is a lack of data on blind adults, the aim of the present research was to make an experimental comparison between the tendency in adults with different visual experience (early blind, late blind and blindfolded sighted) to refer to vision and to the other sensory modalities when freely describing well-known people and familiar haptically-explored objects. The aim was to establish whether or not there was, as could be assumed from models of grounded cognition, any difference in the verbal references to each sensory modality in congenitally blind, late blind and sighted people. We asked three groups of adults (congenitally blind, late blind and blindfolded sighted) to perform two successive and very different tasks. The first one was purely verbal. In it, the participant was asked to describe his/her mother or the woman who took care of him/her, and then his/her father or the man who took care of him/her. Mental representations, prior knowledge and memory processes were involved in the description of these familiar people. Knowledge about these people comes partly from direct perceptive and social experience, and partly from information communicated verbally by the mothers and fathers themselves, and by other familiar people.

The second task was both verbal and haptic. In it, four familiar but unknown objects (a mobile telephone, a toothbrush, a wallet and a bunch of keys) were presented successively one by one, and the participant was asked to describe this object during its manual exploration. In this task, the verbal description was based only on information taken directly from immediate non-visual perceptual processes in association with already available mental representations. Since more words referring to sensory experiences would be produced in the descriptions of unknown objects during perceptual encoding than in the descriptions of known familiar people, this verbal-haptic task would make comparisons between references to each sensory modality possible. More importantly, in the “Objects task”, information about particular unknown properties of the object presented could be gained only through direct non-visual perceptual experience, whereas in the verbal “People task”, a number of the mothers’ and fathers’ characteristics would have been obtained through prior social exchanges with sighted people. Visual references made by the congenitally blind in the “Object task” would therefore have a particular significance for verbalism.

## Methods

### Participants

Thirty adults (15 women and 15 men) living in Grenoble, Lyon or Paris were divided into 3 groups: 10 totally congenitally blind CB (or with only light perception), 10 late blind LB (total blindness from the age of 6, for at least 5 years) and 10 blindfolded sighted (BS) adults. Their ages ranged between 17 and 78 years. The CB and BS groups were matched on age and socio-educational level, whereas the LB group was slightly older and its socio-educational level was slightly lower than that of the two other groups. Table 1 shows the age, age at blindness onset and the socio-educational level of all participants. All participants were monolingual French adults. None of them had any neurological or psychopathological disease. The present research was conducted in accordance with the Declaration of Helsinki and with the norms of the local ethics committee of the LPNC (CNRS and University of Grenoble). All participants gave their informed consent and agreed to the audio-recording of their answers.

**Table 1:** Characteristics of the participants of each group.

Congenitally Blind -CB				Late Blind -LB				Blindfolded sighted - BS		
Age	Sex	Cause of blindness	Educational level	Age	Sex	Cause of blindness	Educational level	Age	Sex	Educational level
27	M	Rétinoblastoma	High school	48	F	CataractAge at blindness: 44	College	22	F	University
25	F	Pigmentary retinopathy	University	78	M	AccidentAge at blindness: 39	Elementary school	26	M	University
23	F	Unspecified	University	31	F	LeukemiaAge at blindness: 8	High school	21	M	High school
17	M	Lebert retinopathy	High school	59	M	Pigmentary retinopathyAge at blindness: 44	Elementary school	26	M	University
36	F	Unspecified	University	70	M	ScotomaAge at blindness: 62	Professional learning	18	M	High school
31	F	Pigmentary retinopathy	University	67	M	Pigmentary retinopathyAge at blindness: 61	University	22	F	University
27	F	Unspecified	University	58	H	Cataract - 9	University	30	F	University
34	M	Unspecified	University	39	F	AccidentAge at blindness: 15	University	31	F	University
29	M	Retina detachment	High school	59	F	Pigmentary retinopathyAge at blindness: 6	High school	30	F	University
31	F	Lebert amaurosis	University	48	M	GlaucomaAge at blindness: 16	High school	31	M	University

### The Tasks

Participants were tested individually in a quiet room, and those who were sighted were blindfolded. All of them were asked to perform two tasks successively. In the first purely verbal one (called the *People Task),* participants were first asked to “describe in 3 minutes your mother or the woman who took care of you”. The same question was then asked concerning “your father or the man who took care of you” (3 minutes). Then, the participant was asked to recount, in 3 minutes, “how your mother or the woman who took care of you could be distinguished from other people”. The final question asked participants to recount, in 3 minutes, “how your father or the man who took care of you could be distinguished from other people”. In the verbal task, the duration of the verbal productions of each participant was, therefore, 12 minutes (4 questions×3 minutes each).

**Figure 1:**
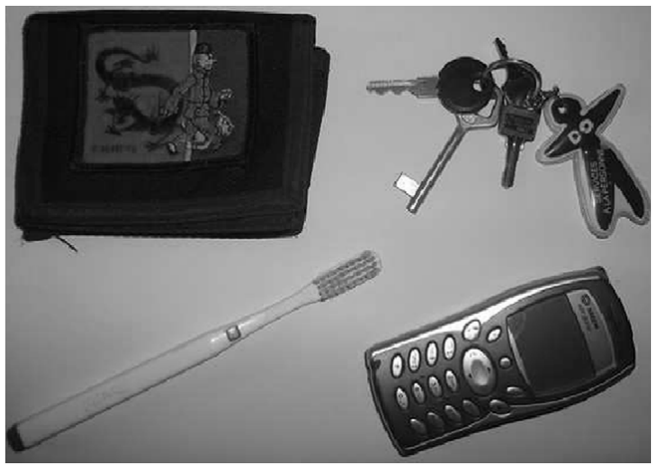
The four objects presented to participants: wallet, bunch of keys, toothbrush, mobile telephone.

The second task (labelled *Objects Task*) was both verbal and haptic. The experimenter presented successively, one by one, four very familiar objects: a toothbrush, a mobile telephone, a bunch of keys and a wallet with a Velcro fastener (Figure 1). For each object, the participant was asked “to perceive (touch) and describe the object in 3 minutes”. The 4 objects were presented in random order. Like the first verbal task, this second task lasted 12 minutes (4 objects×3 minutes each).

In order to avoid the perceptual “Objects task” acting as a prime for touch and audition on the more cognitive “People verbal task”, all participants performed the verbal People task first, followed by the verbal-haptic Objects task. Their verbal productions were recorded in both tasks.

### Scoring and Statistical Analyses

We calculated the total number of significant words (including grammatical operators such as pronouns, adverbs, etc.) produced by each group in each task, and the number of words referring to a sensory modality: vision, touch, audition, olfaction and taste. We added the category of multisensorial words (example of a multisensorial word: *television*, which is both visual and auditory). As far as words implying audition were concerned, we observed that a number of these concerned verbal communication (“she *says*”, “he *explains* that…”, etc.). These words referring to language were not taken into account and only the true sensory references to audition were included in the results (examples: “*her voice reassures me”*, or *“she has her own sounds”).* The most frequently used sensory words are for *Vision*: all the colors, light, transparency, brightness, sighted, blindness; for *Touch*: leather, iron, fabrics, plastic, roughness, smooth, heavy, to hold manually, solidity, relief, Braille; for *Audition*: sounds, music, audiorecord, voice, deafness, bip, cry, to hear; for *Olfactory and Taste:* words were very rarely mentioned and were always related to eating and cooking. For *Multisensory words*, this category was sometimes hard to define because all objects are, in fact, multisensorial. Two main criteria were used: 1. the dominant modalities to which a word refers. Examples: scratch and Velcro (tactile and auditive), television (visual and auditive) decoration and embroidery (visual and tactile); 2. the context of the statement. Example: “something seems to be *written* here but I do not know what/I am unable to say what” (tactile and visual). Written transcriptions of the audio recordings were analyzed by two independent experimenters. The inter-rater agreement was.89. The rare discrepancies observed concerned only multisensory words and were resolved after discussion and redefinition of this category of reference.

In order to analyze count data (number of word), we used PROC GENMOD to fit Poisson error distribution. Due to the repeated nature of the data obtained from each participant (Task; Sensory Modalities), parameters were estimated using the Generalized Estimating Equations (GEEs) approach [22]. GEE methodology is a popular technique for the modeling of correlated response data that are either the result of repeated measurements on the same participant (longitudinal data), or on participants who share the same category that led to correlation [23]. Here, the main hypotheses being tested were whether the group (CB, LB, BS), the task (People, Object) and the sensory modalities (vision, touch, audition, olfaction, taste and multisensory) affected the number of sensory words produced by participants. As olfaction and taste words were very infrequent and often completely absent, we removed these sensory modalities from our statistical analyses. In order to analyse the percentages of sensory words in relation to the number of words produced in each task and group, we used PROC MIXED with repeated data per participant. We applied pre-planned comparisons to test specific contrasts, and post hoc tests for pairwise multiple comparisons (Tukey-Kramer). All statistical analyses were performed using SAS v.9.2.

## Results

### Quantitative Analysis

Preliminary analyses showed that in the People task, answers to the second question (“how can your mother (or your father) be distinguished from other people”) were poor and consisted generally of a repetition of answers to the first question. Therefore, in the following analyses of results, the two questions pertaining to descriptions of participants’ mothers and fathers were combined.

#### A) Total number of words, number of sensory words

Table 2 shows the mean number of total words, the mean number of sensory words and their percentages in relation to the total number of words produced by the congenitally blind (CB), late blind (LB) and blindfolded sighted (BS) participants in each task (description of People and description of Objects). The total number of words revealed no significant main effects of group [Chi2 = 1.41, ddl = 2; p = 0.49], of task [Chi2 = 1.49, ddl = 1; p = 0.22], and no interaction effect between these factors [Chi2 = 0.92, ddl = 2; p = 0.63].

**Table 2:** Mean number of words (and SD), mean number of sensory words (and SD) and percent of sensory words (and SD) relative to the total number of words in the congenitally blind (CB), the late blind (LB) and the blindfolded sighted (BS) groups and in the People and Objects tasks.

Task	Group	Total words	Sensory words	Percent Sensory/Total
**People task**				
	**CB**	858.8±599.0	20.3±17.0	2.2±1.1
	**LB**	922.3±381.2	11.9±12.4	1.1±0.8
	**BS**	733.8±507.4	6.3±6.0	0.8±0.6
**Object Task**				
	**CB**	875.6±562.5	37.6±29.0	3.6±1.6
	**LB**	831.4±322.3	34.2±16.3	4.1±1.3
	**BS**	637.7±363.0	27.3±16.8	4.5±2.0

Regarding the percentages of sensory words in relation to the total number of words produced in both tasks, analyses (PROC MIXED) showed no main effect of group [F(2,27) = 0.30; p = .74], but a significant effect of task [F(1,27) = 62.12; p<.0001]. The percentage of sensory words produced by participants was higher in the Objects task (M = 4.1 with a 95% Confidence Limit [3.58–4.59]), than in the People task (M = 1.3 with 95%CL [0.83–1.84]). The interaction between Group and Task was significant [F(2,27) = 3.83; p = 0.034]. Pairwise multiple comparisons showed that the percentage of sensory references produced verbally in the People task was higher in the CB group (M = 2.18 with 95%CL [1.31–3.05]) than in the LB (M = 1.06 with 95%CL [0.18–1.93]; p = .074) and BS groups (M = 0.79 with 95%CL [0.08–1.66], p = .029). The two latter groups did not differ (p = .66). By contrast, no significant differences were observed between the three groups in the Objects task [all p>.14].

#### B) Number of words referring to each sensory modality

Table 3 shows the mean number of words referring to each sensory modality (vision, touch, audition, multisensory, olfaction and taste) and their percentages in relation to the total number of words produced by each group (Congenitally Blind, Late Blind and Blindfolded Sighted) in each task (Persons or Objects). Globally, references to olfaction and taste were very low and often completely absent. Therefore, the next statistical analyses concerned only words referring to vision, touch, audition, and multisensory words.

**Table 3:** Mean number of words (and SD) referring to each sensory modality and their percents relative (and SD) to the total number of sensory words produced in the congenitally blind (CB), the late blind (LB) and the blindfolded sighted (BS) and in the People and Objects tasks.

Task	Group	Vision	Touch	Audition	Multisensory	Olf.	Taste
**People task**							
	**CB**	7.9±6.5(52.1±28.8)	2.5±2.7(11.3±17)	7.3±7.4(26.5±18.7)	0.8±1.9(2.1±4.5)	0.7	1.1
	**LB**	6.8±5.1(78.9±27.3)	2.4±4.5(8.4±13.4)	1.3±2.7(5.4±11.7)	0.1±0.3(0.3±0.9)	0	1.3
	**BS**	3.6±4.0(49.2±37.2)	0.1±0.3(0.7±2.4)	1.4±1.9(12.7±20.6)	0.5±0.8(4.8±8.7)	0	0.7
**Object Task**							
	**CB**	4.8±6.1(10.4±9.8)	19.2±14.6(47.1±19.6)	2.6±3.4(4.7±5.8)	11±9.7(27.8±18)	0	0
	**LB**	4.5±3.3(14.6±11.7)	19.7±13(55.4±16.2)	1.6±2.4(3.9±6.3)	8±3.4(25±9.9)	0.1	0.3
	**BS**	3.2±3.7(9.4±8.8)	18.1±10.7(65.5±10.2)	0.5±1.3(0.9±2.2)	5.9±3.2(24.2±2.2	0	0

The findings for the number of words referring to a sensory modality revealed no main effect of group [Chi2 = 3.69, ddl = 2; p = 0.16 with M_CB_ = 4.53 95%CL [2.72–7.55], M_LB_ = 3.25 95%CL [2.09–5.04] and M_BS_ = 2.14 95%CL [1.31–3.50]]. The main effects of task and sensory modality were both significant [respectively Chi2 = 11.64, ddl = 1; p<.001 and Chi2 = 13.0, ddl = 3; p<.01]. Sensory words were produced 3.15 times (Odds Ratio = 3.15, 95%CL [2.24–4.44) more frequently in the Objects task than in the People task. Pairwise multiple comparisons showed that sensory references to touch (M = 5.29 95%CL [3.66–7.71]) and to vision (M = 4.74 95%CL [3.49–6.44]) did not differ, but were more frequent than references to multisensory words (M = 1.85 95%CL [1.11–3.08]) or than references to audition (M = 2.14 95%CL [1.36–3.39]). The latter two sensory modalities did not differ, p = .15].

The interaction between Group and Task factors was significant [Chi2 = 7.9, ddl = 2; p = 0.019]. Irrespective of group, the number of sensory words pronounced to describe the Objects task was always at least twice as large (OR_min_ = 2.02) as the number used in the People task.

**Figure 2:**
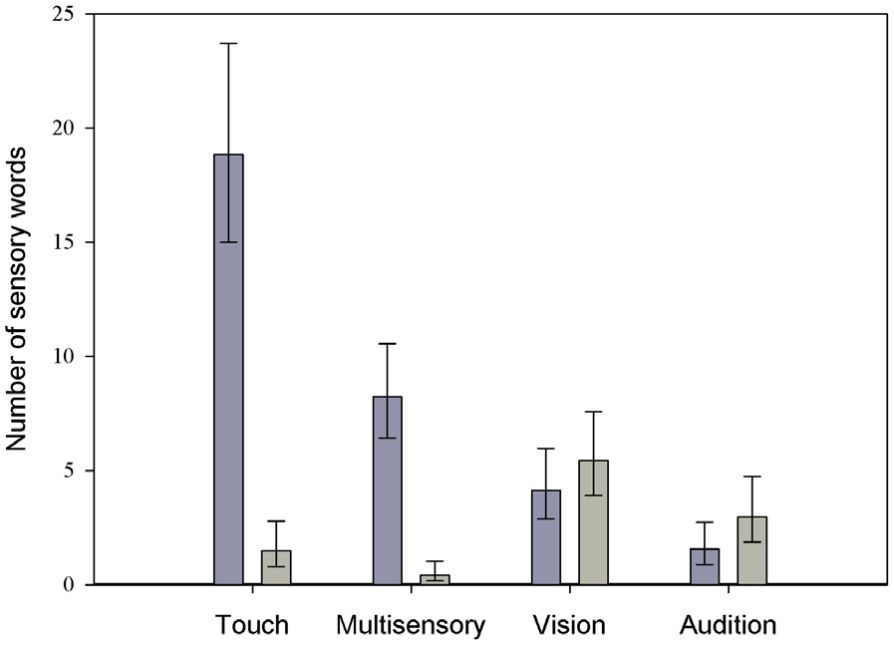
Number of words referring to a sensory modality (touch, multisensory, vision and audition) according to task. The dark gray bars correspond to the Objects task and the light gray bars to Person task. Error bars represent 95% confidence intervals.

The interaction between Task and Modality was significant [Chi2 = 18.91, ddl = 3; p<.001; Figure 2]. In the People task, references to vision dominated and occurred 4.4 times more frequently than references to any other modality [Chi2 = 15.6, ddl = 1; p<.0001]. We found a hierarchy in the frequency of use of the other three modalities, with references to audition being more frequent than references to touch (OR = 1.25 95%CL [1.01–1.57]; Chi2 = 4.18, ddl = 1; p = .041), which was referred to more frequently than multisensory words (OR = 1.52 95%CL [1.13–2.07]; Chi2 = 7.45, ddl = 1; p<.001). In the Objects task, we found a highly significant linear trend (OR for linear trend across modalities = 2.57 95%CL [2.11–3.14]; Chi2 = 20.92, ddl = 1; p<.0001; Figure 2). However, in contrast to the People task, the following modalities were observed in decreasing order of frequency: references to touch, to multisensory words, to vision and to audition.

The interaction between Group and Modality was not significant [Chi2 = 10.4, ddl = 6; p = .109]. Nevertheless, one finding does deserve to be highlighted: in the CB group the number of references to audition was 3.91 times greater than in the LB and BS groups [Chi2 = 9.58, ddl = 1; p<.01]. Finally, the triple interaction between Group, Task and Modality was not significant [Chi2 = 10.8, ddl = 6; p = .095].

### Qualitative Analysis

Previous quantitative analyses revealed that the total number of sensory words was relatively low in all groups. Qualitative analysis showed that in the People (descriptions of the mother and the father) task, most verbal references concerned the personality, the professional activity, the social and affective relationships between participants and these people and, for blind participants, how their childhood visual impairment was accepted by their parents. Examples: *“dynamic but very stressed*”, “*he/she is someone who reassures us when we have a problem”*, *“he accepted my handicap easily, and he coped well with difficult situations, etc*.” In the Objects task, the first verbal productions of participants were always devoted to the search for cues which would enable identification of the object and, once identification had taken place (with no errors on the part of any of the participants), the comments mainly concerned the use of these objects and their practical interest in everyday life. Examples: “*this small pocket (in the wallet) is for the money and this is for bank cards”*, “*it’s not easy for me to use”,* “*this mobile phone is an old one*”, etc.

### Discussion

The aim of this research was to study the relative weight of verbal references to the different sensory modalities in descriptions of people and objects by three groups of adults with different visual experience: congenitally blind (CB), late blind (LB) and blindfolded sighted (BS). The literature had previously revealed a tendency of the CB to refer to visual attributes of which they have no perceptual experience (“verbalism”) while conversely; references to touch and audition in their verbal productions were rare, even though these modalities are systematically used in blindness. However, in the light of new data, this description of the oral language of the blind had been questioned recently. For example Eardley and Pring [24] did not observe a difference in the visual and non-visual imagery of congenitally blind and sighted adults in a task of autobiographical memory, whereas in a generation of future task (imagining the future), the CB evoked more auditory images than the sighted. In addition, in the context of recent theories claiming that cognition is not amodal but is firmly linked to the perceptions and actions from which it derives [4], it may be assumed that the specific perceptual experiences of blind people will lead them to use more sensory words referring to the sensory modalities they use in everyday life. The weight of the different sensory modalities in their verbal descriptions could, therefore, differ from the verbal productions of sighted people.

We recorded the verbal descriptions of well-known people (mother and father, Persons task) and of concrete, manually-explored, familiar objects (Objects task) in congenitally blind (CB), late blind (LB) and blindfolded (BS) adult participants, and we calculated the number of verbal references to each sensory modality in each task and each group.

Our results showed firstly that the total number of words produced within the limited time of 12 minutes did not differ according to the tasks and the groups, although inter-individual differences were high. Verbal fluency was therefore the same in all groups and conditions. This observation is in line with Eardley and Pring [24] who found no difference between congenitally blind and sighted adults in a specific test of verbal fluency. This means that early blindness does not increase the rate of verbal productions, at least in the circumstances tested in these studies.

Another analysis of our results showed that the percentage of sensory words (words referring to a sensory modality) was low compared to the total number of words produced, and that there was no main effect of group. However, in the People task, the CB group produced more sensory words than the LB and BS groups. Finally, as predicted, more sensory references were obtained in descriptions of Objects during perceptual encoding than in descriptions of known People.

The most interesting analyses concerned the number of sensory words referring to each modality in each group and each task. The main effect of task showed that, as predicted, participants produced 3 times more sensory words in the Objects task than in the People task. The main effect of modality revealed no difference between the number of references to vision and to touch, whereas both these modalities were consistently more frequently used than multisensory and auditory words, which did not differ. Therefore, vision and touch were the most often cited sensory modalities. However, although the interaction between group and modality was not significant, results showed that the CB referred to audition quite four times more often than the other two groups. The particular emphasis on audition resulting from early blindness does therefore appear in the verbal productions of the CB.

Another significant result concerned the interaction between modalities and tasks. In the People task, visual references were clearly dominant (they were produced 4.4 more often than the other modalities). Although lower than visual references, auditory words were more frequent than tactile words which in turn were more frequent than multisensory words. By contrast, in the Objects task, this hierarchy was reversed. Touch was the most frequently cited sensory modality, followed by multi-sensory words, while the percentage of references to vision and audition was extremely low. This means that the hierarchical order of the sensory modalities is not fixed, but varies according to the situation. Touch was maximally evoked during perceptual encoding of objects whereas vision (and audition) dominated during descriptions of people. By contrast, in the Objects task, there were no differences between groups: references to touch dominated clearly across the board, whereas references to vision and audition were scarce.

To sum up, this study confirmed that congenitally blind adults evoked visual words just as frequently as the sighted. It seems therefore that for the CB, the visual attributes of people (mainly the colour of eyes, hair and sometimes clothes) constitute important cues, even though the early blind have no direct experience of these cues, and have gained information about them only through verbal exchanges with sighted people. This result appears to support the conclusion of the study conducted by Rosel, Caballer, Jara and Oliver [12], suggesting that as the child grows and progressively integrates all aspects of adult language, his own verbal communication becomes less specific to blindness and increasingly similar to that of sighted people, especially where visual sensory references are concerned. However, this tendency appeared only in descriptions of People and, in this task, more references to audition were observed in the CB than in the LB and the BS groups. The specific perceptual experience of the congenitally blind did therefore manifest itself in their verbal productions.

By contrast, when describing manually-explored, concrete objects, references to touch and to multisensory words (mainly tactile + auditory) dominated in all groups, whereas references to vision were scarce. This means that the relative weight of each sensory modality varies according to the cognitive processes involved in each task: the long term memory and internal representations at work in the People task seem to favour visual references in both the blind and the sighted, whereas the congenitally blind also make references to audition. By contrast, the perceptual encoding and working memory involved in the Objects task enhance sensory references to touch equally in all three groups. This result stems from the fact that the description of objects occurred during manual exploration. Future research should examine verbal descriptions of familiar objects which are not presented physically during the task.

In conclusion, this set of results attenuates the impact of verbalism in blind adults since verbalism depends on situation. It also supports (albeit moderately) the idea that the perceptual experience of the congenitally blind adults is to some extent reflected in their cognition.
